# Nanomaterial-Enabled Flexible Wearable Sensors for Sports Monitoring and Rehabilitation: Material Architectures, Interface Design, and Signal Reliability

**DOI:** 10.3390/nano16211367

**Published:** 2026-10-26

**Authors:** Yuanqingqing Tao, Ziyi Hao, Yi Zhang, Guo Li, Baizeng Fang

**Affiliations:** 1 School of Physical Education, Nanjing Tech University, Nanjing 211816, China; taoyuanqingqing@njtech.edu.cn; 2 Faculty of Education, The Chinese University of Hong Kong, Hong Kong, China; 1155254029@link.cuhk.edu.hk; 3 School of Energy Science and Engineering, Nanjing Tech University, Nanjing 211816, China; zhangy@njtech.edu.cn; 4 School of Chemical Engineering and Energy Technology, Dongguan University of Technology, Dongguan 523808, China

**Keywords:** nanomaterials, flexible wearable sensors, body–sensor interfaces, signal reliability, sports monitoring and rehabilitation

## Abstract

Flexible wearable sensors increasingly use nanomaterials and nanoscale interfaces to acquire biomechanical, electrophysiological, and biochemical signals during sports and rehabilitation, where repeated deformation, perspiration, and variable body contact challenge measurement stability. This review examines how the nanomaterial architecture and body–sensor interface design influence signal reliability under dynamic operating conditions. Carbon nanomaterials, MXenes, conductive polymers, metallic nanostructures, and soft nanocomposites are discussed together with matrices and fibrous supports that regulate their mechanical and chemical interfaces. Emphasis is placed on the links between nanoarchitecture, transport behavior, interfacial coupling, and major transduction routes. Representative applications in motion monitoring, exercise physiology, sweat analysis, and rehabilitation are then assessed in terms of signal stability and physiological interpretability. The literature indicates that sensitivity alone is an incomplete measure of wearable performance because working range, hysteresis, adhesion, sampling variability, and calibration drift often become limiting factors under repeated use. Reliable sensing depends on preserving a stable relationship between the target stimulus and sensor output across realistic operating conditions. Future work should focus on robust nanoarchitectures, adaptive body–sensor interfaces, application-specific calibration, and validation under combined mechanical and environmental loading.

## 1. Introduction

Flexible wearable sensors have extended biomechanical and physiological measurements from laboratory settings to continuous on-body monitoring. Epidermal electronics, stretchable electrochemical devices, textiles, and soft microfluidics reduce mechanical mismatch with the body and permit measurements during natural movement [1,2,3,4,5]. Sports and rehabilitation impose particularly demanding conditions because large joint excursions, repetitive loading, perspiration, and repeated attachment continually modify the body–sensor interface [6,7,8]. Parashar et al. developed a self-powered triboelectric insole using nylon nanofibers and microstructured PTFE layers for gait analysis and rehabilitation monitoring, demonstrating stable sensor responses under varying humidity and temperature conditions [9]. Reliable measurement in these settings requires a stable relationship between the target physiological or biomechanical variable and the recorded signal.

This requirement is not captured by isolated figures of merit. Near-threshold conductive networks may produce large resistance changes but can also narrow the usable strain range or increase hysteresis, while hydrated ionic materials remain sensitive to temperature and water content. Electrochemical measurements add further uncertainty through sweat generation, transport, and reference electrode stability. Gao et al. demonstrated multiplexed sweat analysis during on-body operation [10], Alizadeh et al. monitored electrolytes during exertion [11], and Parrilla et al. examined validation requirements for wearable ion sensing [12]. Work on sweat lactate likewise showed that analytical sensitivity does not by itself establish physiological relevance [13], whereas multiparameter measurements can provide the context needed for interpretation [14]. Material response, interface stability, sampling, and transduction, consequently, have to be considered within the same measurement chain.

The material basis of wearable sensing spans conductive nanostructures, electrochemically active surfaces, and compliant matrices. Carbon nanotubes and graphene use nanoscale contact reconstruction and tunneling for mechanical sensing [15,16,17], while MXenes combine high electronic conductivity with hydrophilic, chemically active surfaces [18,19,20]. Singh et al. reviewed the applications of two-dimensional nanomaterials in biomarker sensing and neural bioelectronic interfaces, highlighting the importance of material properties and biological interactions in interface design [21]. Conductive polymers and metallic nanostructures offer tunable charge-transfer interfaces [22,23,24]. Hydrogels, nanofibers, and textiles mainly serve as compliant hosts that control dispersion, strain transfer, ionic transport, adhesion, breathability, and fluid handling [8,25,26,27,28]. Performance thus depends on how nanoarchitecture and surface chemistry interact with the mechanics and environment of the body–sensor interface, not on composition alone.

**Figure 1:**
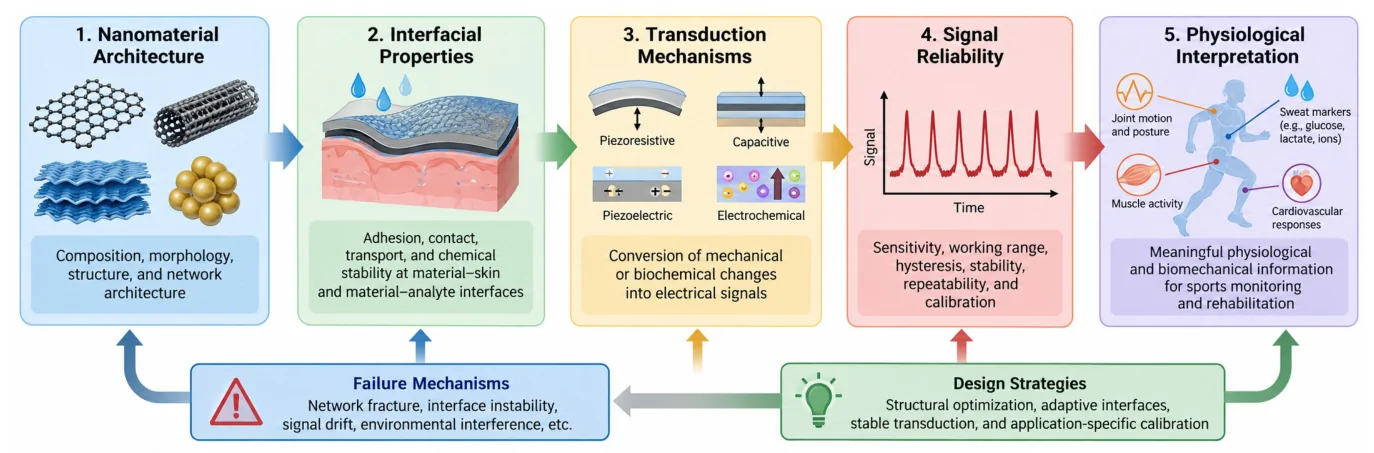
Conceptual framework linking nanomaterial architecture, interfacial properties, transduction mechanisms, signal reliability, and physiological interpretation in wearable sensing for sports monitoring and rehabilitation, together with associated failure mechanisms and design strategies.

In practical wearable sensing, the effects of material composition and nanoarchitecture are closely linked to interfacial interactions and the conditions under which sensors operate. Previous reviews have examined sports wearables, electronic textiles, hydrogel sensors, and continuous molecular monitoring, providing valuable insights into material development, device design, and application requirements [3,29,30,31]. These studies provide a foundation for understanding wearable sensing technologies, while the combined influence of nanoscale architecture and interfacial chemistry on transduction stability and signal reliability merits further consideration. Accordingly, this review examines how material properties and interface behavior affect sensing mechanisms and the interpretation of biomechanical and physiological signals. Major nanomaterial families and their supporting matrices are discussed first, followed by an analysis of the factors governing signal transduction. Representative applications in sports and rehabilitation are then examined in relation to practical challenges, including repeated deformation, perspiration, sensor attachment, and inter-individual variability. This discussion connects material design with sensing reliability and physiological interpretation under realistic wearable conditions. Figure 1 summarizes these relationships and illustrates the associated failure mechanisms and design strategies across the wearable sensing process.

## 2. Nanomaterial Architectures and Interface Design for Dynamic Wearables

Nanomaterial-enabled wearables translate nanoscale changes in contact, spacing, surface state, or molecular interaction into electrical signals while the device is continuously deformed and exposed to the body environment [32]. The decisive design variables therefore extend beyond chemical composition to include dimensionality, junction density, porosity, interfacial bonding, and the manner in which active nanostructures are embedded in a compliant host. Four coupled attributes are particularly important: continuity of electronic or ionic pathways, mechanical compliance and strain transfer, interfacial adhesion, and resistance to environmental change. The following comparison emphasizes how nanoarchitecture and interface chemistry jointly regulate these attributes, rather than treating conductivity, stretchability, or sensitivity as independent material properties.

### 2.1. Carbon Nanomaterials: Percolation, Tunneling, and Hierarchical Networks

Carbon-based strain sensors establish network reconfiguration as a primary electromechanical mechanism. CNT-film studies linked processing-dependent morphology with flexible electronic behavior [33]. Yamada et al. used aligned SWCNT films in stretchable human-motion sensors [17], and Wang et al. later employed graphene woven fabrics to amplify small deformations through junction-resistance changes [15]. These studies showed that useful sensitivity arises from controlled changes in contacts and tunneling distances rather than from high bulk conductivity alone.

The same mechanism also explains a major limitation: sensitivity increases as a network approaches electrical disconnection. Shi et al. reinforced CNT networks with graphene to improve load transfer and cyclic stability [16], whereas Boland et al. showed that viscoelastic graphene-polymer matrices introduce time-dependent relaxation into large resistance changes [34]. Graphene-foam/polyurethane composites further demonstrated that matrix-supported connectivity improves recovery [35]. A practical carbon network must therefore permit reversible rearrangement while retaining enough redundant pathways to suppress irreversible drift.

Recent work has consequently shifted toward wider deformation windows and wearable robustness. Wang et al. introduced a waterproof, breathable graphene electronic fabric [36], while Yoon et al. combined rGO with laser-induced graphene to span subtle physiological deformation and large joint motion [37]. Architecture-based comparisons of graphene films, fibers, foams, and hydrogels reinforce the same conclusion [38]: carbon materials are best viewed as configurable network elements, and their performance should be matched to strain amplitude, loading history, and environmental exposure rather than ranked by conductivity alone.

### 2.2. MXenes and Other Two-Dimensional Nanomaterials

MXene sensing emerged from the chemistry of layered carbides rather than from a sensor-specific discovery. Naguib et al. reported exfoliated ${\mathrm{Ti}}_{3}$$C_{2}$ in 2011 [39], Ghidiu et al. subsequently introduced conductive ${\mathrm{Ti}}_{3}$$C_{2}T_{x}$ “clay” [40], and Anasori et al. consolidated the broader MXene materials framework [41]. Work on ultrathin two-dimensional materials similarly established thickness, surface chemistry, and interfacial transport as key nanoscale design variables [42]. Metallic conductivity, hydrophilic terminations, and interlayer water support ion access and functionalization, but they also make restacking, oxidation, and hydration state intrinsic sources of signal variability.

**Figure 2:**
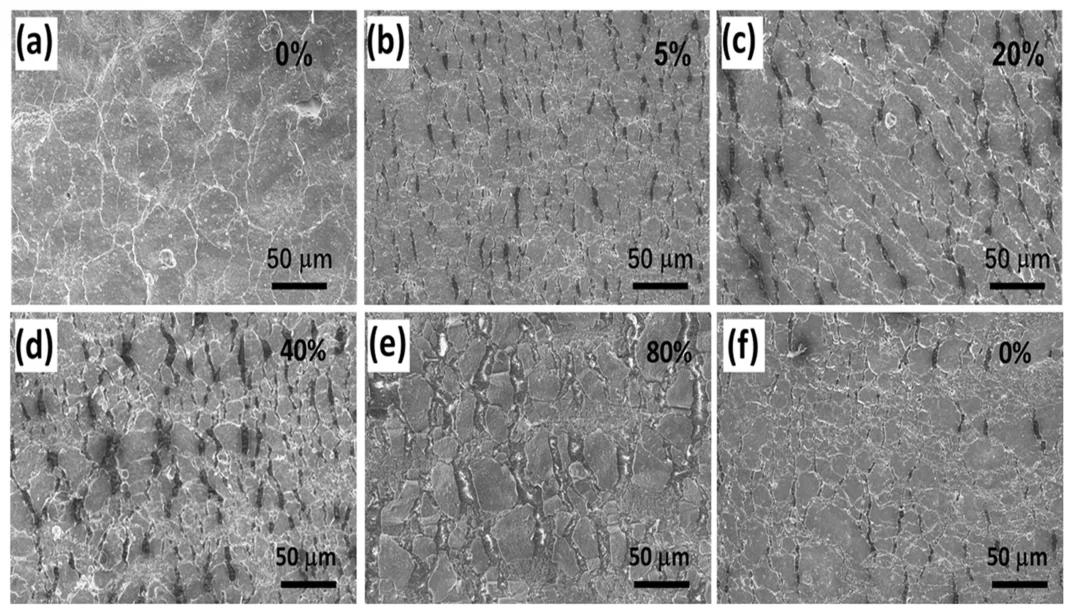
Strain-induced crack evolution in the ${\mathrm{Ti}}_{3}$$C_{2}T_{x}$/CNT conductive network at different tensile strains: (**a**) 0%; (**b**) 5%; (**c**) 20%; (**d**) 40%; (**e**) 80%; and (**f**) recovery after unloading to 0% strain. Adapted from Ref. [43].

Wearable devices translated these layered properties into both mechanical and electrochemical transduction. Cai et al. bridged ${\mathrm{Ti}}_{3}$$C_{2}T_{x}$ nanostacks with CNTs so that current pathways survived strain-induced cracking [43]. Figure 2 shows the progressive opening of cracks in the MXene layer with increasing tensile strain while CNT bridges preserve conductive connections across the separated domains. This directly visualizes how nanoscale crack evolution can be converted from an uncontrolled failure process into a reversible piezoresistive mechanism. Porous-paper and textile-supported MXene networks then extended pressure sensing to compliant substrates [19,44]. In parallel, enzyme immobilization [45], charge-transfer studies [46], and sweat analysis [20] established MXene as an electrochemical interface. These two branches exploit the same surface-rich material but require different stability criteria: mechanical sensors depend on reversible contact evolution, whereas biochemical sensors additionally require chemically stable surface states.

As MXene wearables matured, interfacial stability became as important as initial conductivity. Reviews from 2020 onward increasingly linked oxidation, water loss, and restacking to biosensor and pressure-sensor reliability [47,48,49]; a recent ACS Nano review likewise emphasizes surface stabilization and composite design in wearable physical sensors [50]. Zhao et al. used a water-retaining semi-interpenetrating hydrogel to stabilize strain response [51], and Chae et al. suppressed oxidation with a self-healable catechol-functionalized matrix [52]. Dynamic MXene-hydrogel coupling provides a further route to stabilize the interface [53]. The development path therefore moves from exploiting a highly conductive 2D surface to engineering the chemical and mechanical environment that preserves that surface during wear.

### 2.3. Conductive Polymers and Metallic Nanostructures

Conductive polymers entered biosensing before the current generation of soft wearables. Early PANI-based systems established that enzyme coupling, doping state, morphology, and charge transfer jointly determine electrochemical performance [54,55], a relationship later generalized in reviews of polymer–nanocomposite biosensors [56]. Wearable formats retained this chemistry while adding mechanical compliance and ion access. Textile potentiometric pH sensing [57], electrochemically gated organic semiconductors and conjugated-polymer nanocomposites [58,59], and porous PEDOT:PSS/graphene electrodes for sweat glucose and pH [60] illustrate this transition. Gao et al. recently integrated PEDOT, PVA, and MXene into a porous hydrogel for uric-acid sensing [61]. The progression is therefore from a conducting coating toward a multifunctional matrix that coordinates electronic transport, ionic access, and recognition chemistry.

Metallic nanostructures followed a parallel route. Transfer printing and all-printed stretchable electrochemical devices first enabled conductive electrodes on deformable, non-planar substrates [24,62]. Xu et al. then used graphene/PANI/Au nanoparticles to promote glucose-oxidase electron transfer [63] and later integrated Au nanoparticles with PANI arrays on carbon cloth [64]. Bae et al. coupled nanoporous gold with capillary microfluidics for continuous glucose monitoring [22]. These examples show why conductivity or catalytic area alone is insufficient: the metallic phase must remain electrically connected, chemically accessible, and mechanically supported during deformation.

Later polymer/metal hybrids retain the established roles of doping, molecular recognition, and charge transfer, but distribute these functions across deformable composite interfaces. Soft phases accommodate strain and ionic transport, while nanoscale conductive or catalytic domains maintain electrical transduction. Table 1 compares representative systems using metrics appropriate to each sensing modality.

The values in Table 1 should not be used for direct ranking across sensing modalities. Carbon and MXene networks respond mainly through contact or crack evolution, whereas polymer/metal systems depend more strongly on surface chemistry and charge-transfer kinetics. However, changes in polymer doping states and the degradation of metallic interfaces may compromise signal stability during prolonged use [24,54,60]. Gauge factor is meaningful only within its tested strain window, and the detection limit is informative only when sampling and recognition remain stable. Differences in substrates, electrolytes, deformation protocols, and calibration procedures further limit direct numerical comparison between studies.

### 2.4. Soft Matrices, Fibers, and Adaptive Interfaces for Nanomaterial Networks

For skin-mounted wearable sensors, soft matrices and fibrous scaffolds are important not only as mechanically compliant substrates but also as hosts that organize, protect, and mechanically couple active nanomaterials to the body. In this context, hydrogels and textiles are considered primarily for the way they stabilize conductive or ionic pathways, regulate nanomaterial dispersion and interfacial contact, and transfer body deformation to nanoscale sensing elements. Early soft-interface designs focused on reducing mechanical mismatch between the sensing layer and the body. Sun et al. demonstrated a tough double-network hydrogel combining high water content with large extensibility [27], and Keplinger et al. showed that highly deformable hydrogels could function as transparent ionic conductors [28]. Yoon et al. subsequently introduced rapid self-healing into a sweat-monitoring material [69]. Reviews now distinguish ionic from electronic conductive hydrogels and emphasize simultaneous control of mechanics, conductivity, adhesion, and environmental stability [70,71]. These matrices therefore provide a material platform in which nanoscale conductive phases can remain electrically or ionically connected while the macroscopic interface follows skin deformation.

**Figure 3:**
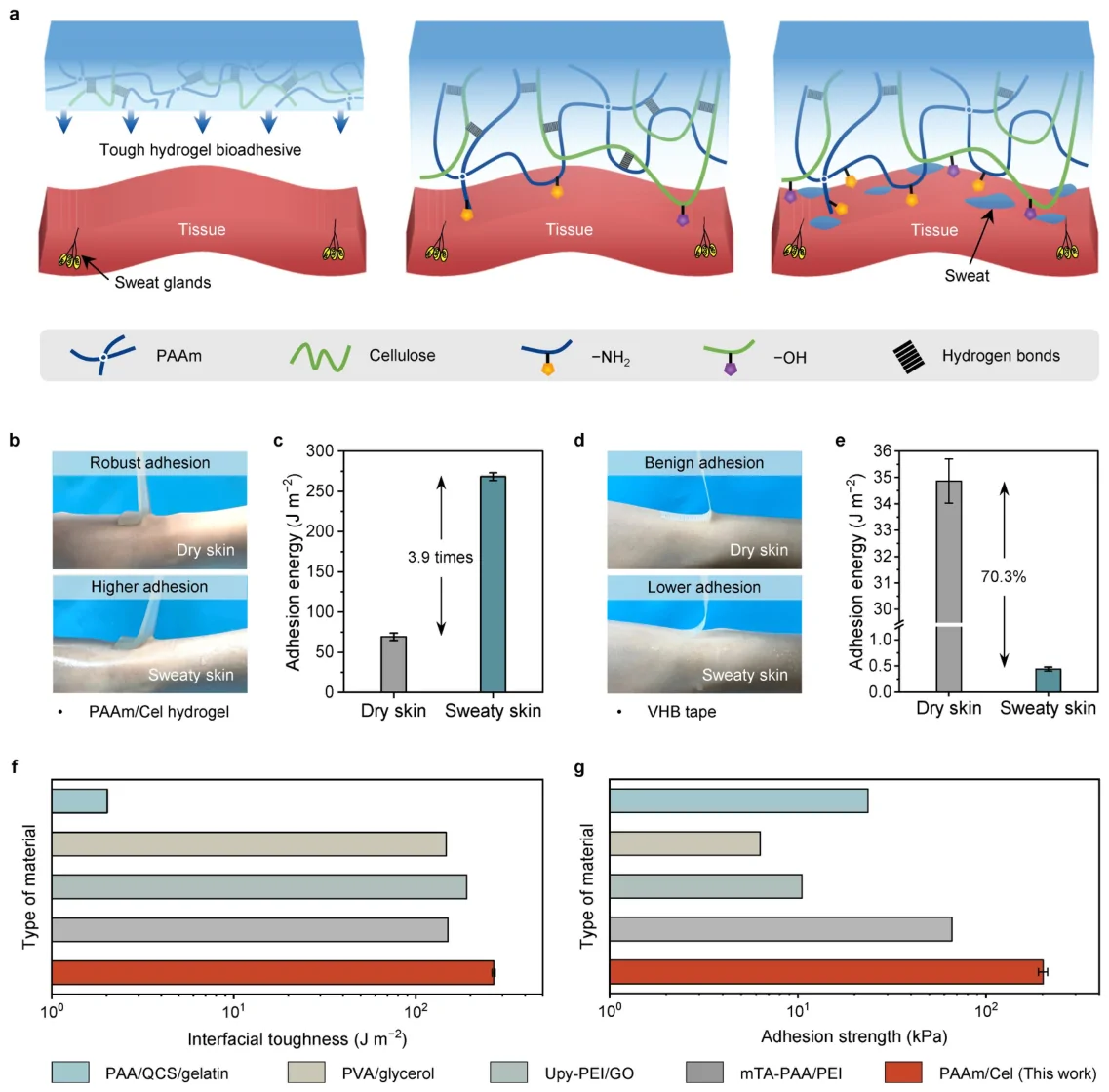
Sweat-adaptive adhesion of the PAAm/Cel hydrogel: (**a**) schematic illustration of dynamic hydrogen-bond regulation at the hydrogel–skin interface before and after sweating; (**b**) adhesion performance of the PAAm/Cel hydrogel on dry and sweaty skin; (**c**) comparison of adhesion energy on dry and sweaty skin; (**d**) adhesion performance of conventional VHB tape on dry and sweaty skin; (**e**) corresponding adhesion-energy comparison for VHB tape; (**f**) comparison of interfacial toughness with representative adhesive materials; and (**g**) comparison of adhesion strength with representative adhesive materials. Adapted from Ref. [75].

Environmental stability and skin adhesion introduce additional constraints. Antidrying organohydrogels use solvent engineering to preserve ionic transport outside ambient conditions [72], and later systems extended this strategy to ultralow temperatures [73]. Lignin-modified MXene hydrogels increased mechanical and environmental tolerance [74], while Wang et al. designed a PAAm/Cel network in which dynamic hydrogen bonds strengthened skin adhesion after sweat exposure [75]. Figure 3 compares the interface before and after sweating and shows stronger adhesion under conditions that weaken conventional adhesive contact. This behavior illustrates how water and ions at the skin surface can be incorporated into interface design. Mesh reinforcement [76], MXene/PVA-acrylic-acid physical crosslinking [77], nanomesh-reinforced eutectogels, and dynamically coupled MXene hydrogels [78,79] address the same stability problem through structural reinforcement and reversible interactions. High stretchability is valuable only when the nanomaterial-matrix–skin interface remains stable during use.

**Figure 4:**
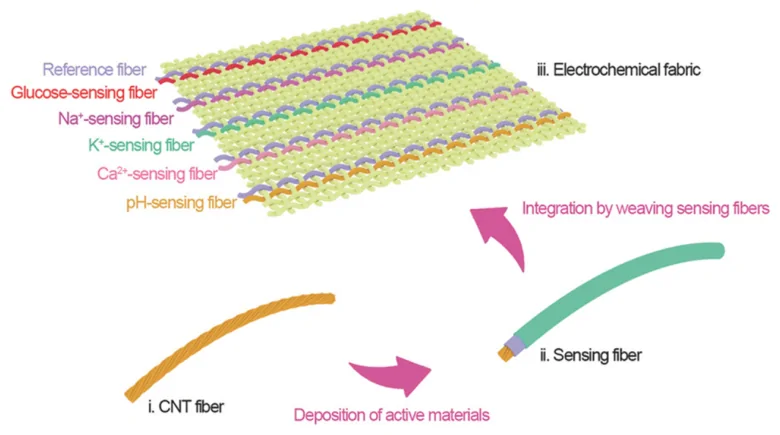
Fabrication of a multifunctional electrochemical fabric by functionalizing and weaving CNT sensing fibers. Adapted from Ref. [84].

Fiber and textile platforms show a related evolution in which the scaffold itself becomes functional. Screen-printed amperometric electrodes and CNT/polymeric-membrane yarns first preserved electrochemical operation during garment deformation [80,81], followed by stretchable textile potentiometry for multi-ion sensing [82]. Reviews of fiber electronics framed this transition from passive support to active device architecture [83]. Wang et al. then wove differently functionalized CNT fibers into a common electrochemical fabric [84]. As summarized in Figure 4, individual CNT fibers are first functionalized for distinct analytes and are then integrated by weaving into a shared textile platform, illustrating how nanoscale sensing interfaces can be modularized at the fiber level without losing garment-level flexibility. Later breathable and washable sensors introduced vapor transport and repeated-use stability as explicit metrics [85], while nanofiber and triboelectric webbing systems integrated strain accommodation, permeability, or energy conversion into the scaffold [26,86]. Textile mechanics must therefore be treated as part of calibration, not merely packaging.

Table 2 compares these systems through matrix chemistry, reversible interactions, interfacial mechanics, and environmental durability. Such descriptors are more informative than sensitivity alone when the design objective is to preserve transport and body coupling through repeated wear.

For wearable applications, the suitability of these materials depends not only on their intrinsic sensing properties but also on the stability of their conductive networks and interfaces during use. Carbon nanomaterials provide flexible conductive networks with high sensitivity, although junction rearrangement and polymer relaxation can lead to hysteresis and signal drift under repeated deformation [16,34]. MXenes offer high conductivity and chemically active surfaces, but oxidation, restacking, and changes in hydration can compromise their long-term stability [48,52]. Conductive polymers enable flexible electrochemical interfaces, while variations in doping state and moisture content may alter their electrical and electrochemical responses [54,60]. Metallic nanostructures provide high conductivity and catalytic activity, but junction damage and interfacial degradation can impair durability [24]. In addition to these performance-related limitations, biocompatibility requires careful assessment of potential nanomaterial release, residual processing chemicals, and metal-ion exposure during prolonged skin contact [89,90]. Soft matrices and encapsulation can help stabilize sensing structures and reduce direct exposure, although they may also restrict breathability and mass transport. Material selection consequently involves balancing sensing performance, environmental stability, skin-contact safety, and fabrication reproducibility. Differences in deformation rate, humidity, sweat exposure, and testing protocols also complicate quantitative comparisons across studies and highlight the need for more consistent evaluation under realistic wearable conditions.

## 3. From Material Structure to Signal Transduction and Device Performance

Device performance is determined by how deformation, interfacial change, or molecular interaction perturbs a measurable electrical quantity. Sensing mechanisms differ in both the type of output produced and the material variable responsible for that output. Their evaluation should therefore include the reversibility and stability of the underlying structural or interfacial change, in addition to sensitivity, operating range, response time, and hysteresis.

### 3.1. Piezoresistive Strain and Pressure Sensing

**Figure 5:**
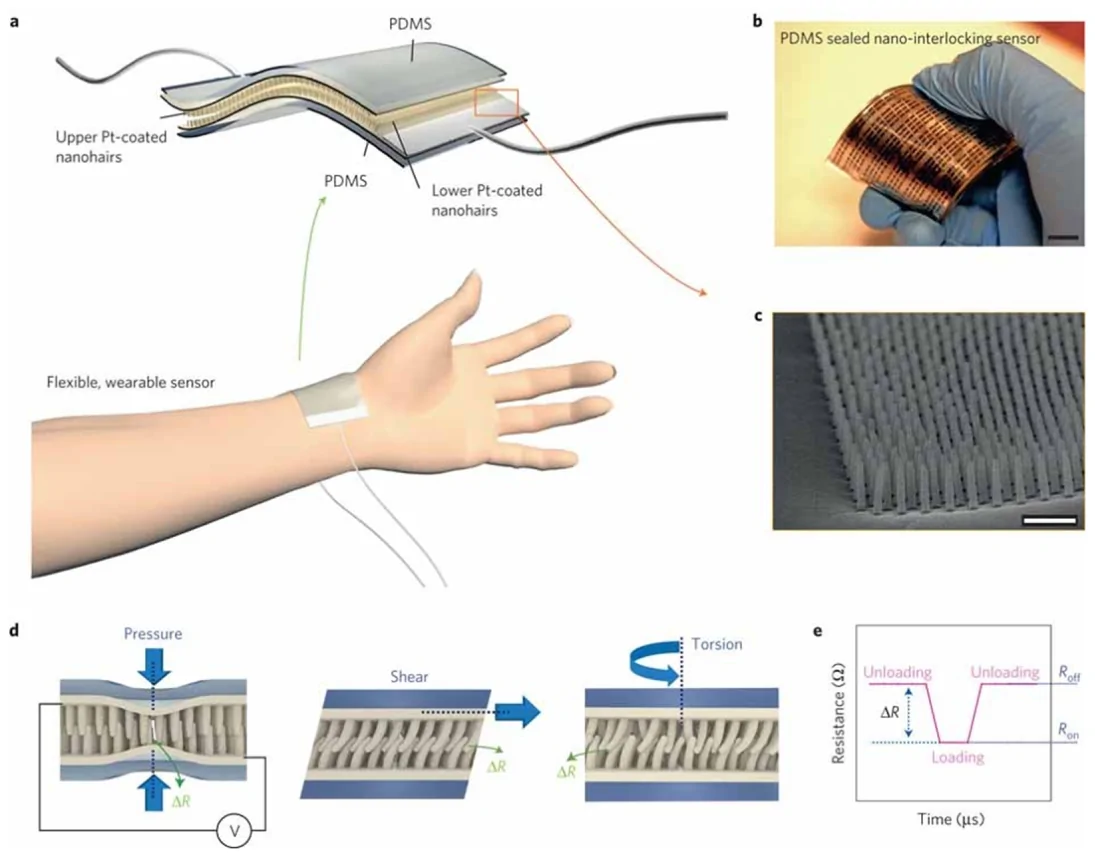
Reversible interlocking Pt-coated nanofiber strain sensor: (**a**) schematic illustration of the flexible sensor structure and wearable configuration; (**b**) photograph of the flexible sensor under bending; (**c**) morphology of the interlocking Pt-coated nanofiber structure; (**d**) schematic responses under normal pressure, shear, and torsional loading; and (**e**) corresponding resistance variation during loading and unloading. Adapted from Ref. [91].

Piezoresistive sensors provide a clear example of how mechanical deformation is translated into electrical resistance through the reorganization of conductive contacts and junctions. Pang et al. used the reversible interlocking of Pt-coated nanofibers to amplify resistance changes through contact reconstruction [91]. Amjadi et al. extended this concept to Ag-nanowire/elastomer networks, where junction sliding and reconnection accommodate larger strains [92], while mechanoreceptor-inspired structures introduced local mechanical amplification [93]. Figure 5 shows the interlocked nanofiber architecture together with its response to normal, shear, and torsional loading. The observed resistance changes follow changes in contact topology, illustrating why nanoscale junction evolution is more informative for this mechanism than bulk conductivity alone.

Later studies addressed the loss of stability that often accompanies highly responsive networks. Viscoelastic graphene-polymer interfaces showed how relaxation contributes to hysteresis [34], whereas ${\mathrm{Ti}}_{3}$$C_{2}T_{x}$/CNT hybrids maintained conduction across MXene cracks through CNT bridging [43]. These results place the gauge factor in context: a large response is useful only when the conductive network can repeatedly reorganize without irreversible disconnection. Working range, hysteresis, and cyclic stability are essential companion metrics for wearable motion sensing.

### 3.2. Capacitive, Iontronic, and Piezoelectric Transduction

Capacitive, iontronic, and piezoelectric sensors generate signals through changes in charge distribution rather than through resistive-network continuity. ZnO nanowire generators established direct mechanical-to-electrical conversion for dynamic deformation [94], while microstructured PDMS dielectrics amplified pressure-induced capacitance changes [95]. Keplinger et al. introduced highly deformable ionic conductors [28], and CNT-based capacitive devices extended geometric transduction to wearable strain sensing [96]. In these devices, dielectric geometry, ionic interfaces, and polarization form part of the active transduction process.

Their relative advantages depend on signal timescale. Capacitive and iontronic devices are suited to sustained geometric changes when spacing and interfacial capacitance remain stable, whereas piezoelectric outputs naturally emphasize impact, vibration, and transient force. More recent piezoelectret systems improve sensitivity for non-contact motion and physiological monitoring [97], but useful performance still requires mechanical and frequency matching to the target movement. Humidity and water content can additionally alter dielectric and ionic properties [98]. Mechanism selection should therefore follow the temporal and environmental characteristics of the application rather than a generic sensitivity ranking.

### 3.3. Electrophysiological Interfaces

Electrophysiological sensing makes the electrode–skin interface a primary determinant of signal quality. Ultrathin epidermal electronics established the importance of conforming to natural skin deformation [4], Imani et al. combined electrophysiological contact with chemical sensing in one wearable platform [99], and stretchable matrix networks extended conformal coupling over larger strains [5]. These studies show that high electrode conductivity cannot compensate for motion-induced changes in contact impedance.

**Figure 6:**
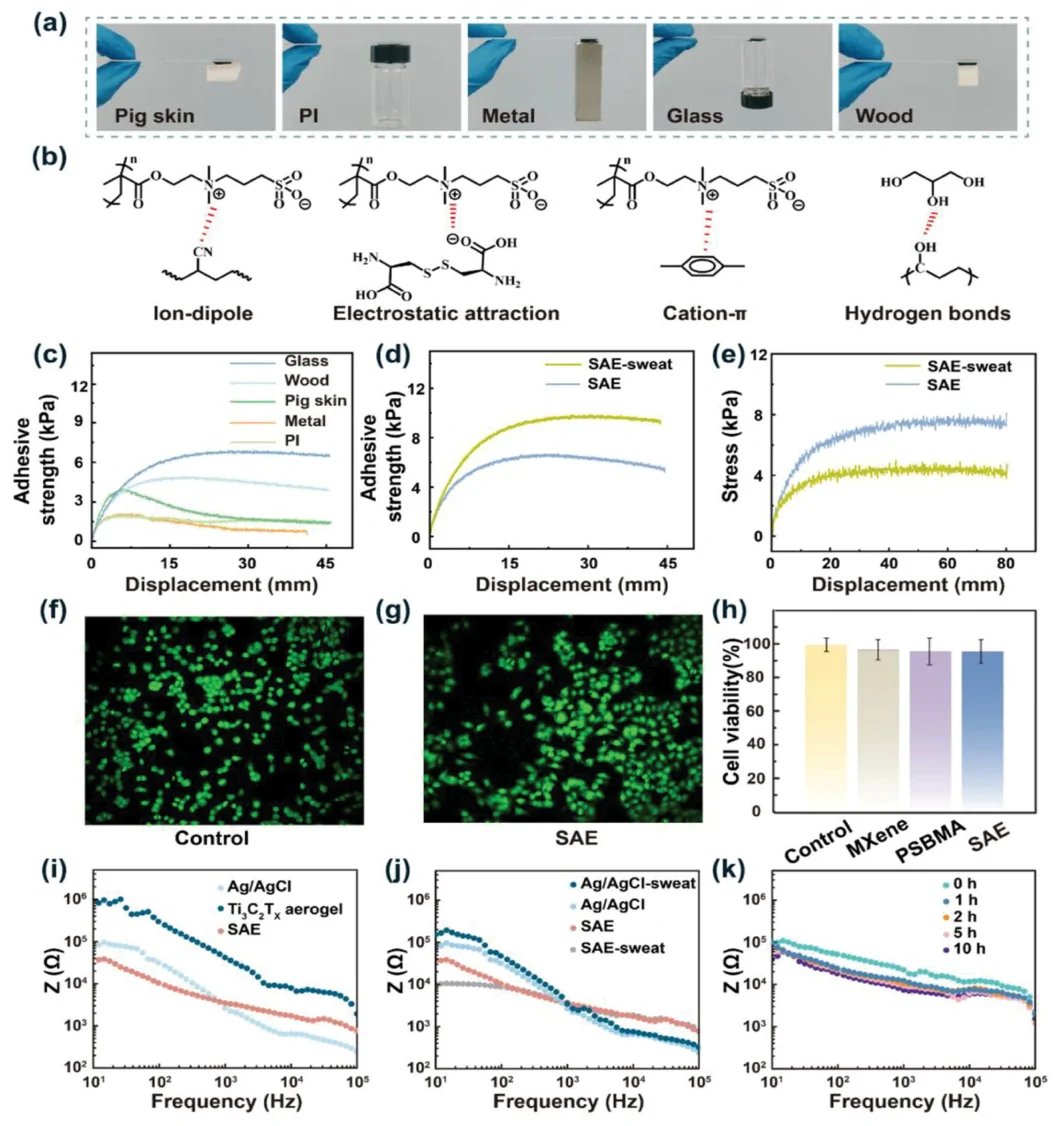
Adhesion, biocompatibility, and skin-contact impedance of the sweat-absorbing skin electrode (SAE): (**a**) adhesion of the SAE to different substrates; (**b**) schematic illustration of the interfacial adhesion mechanisms; (**c**) shear strength of the SAE on different substrates; (**d**) shear strength on glass before and after artificial-sweat exposure; (**e**) adhesion strength before and after artificial-sweat exposure; (**f**,**g**) fluorescence images of HeLa cells in the control and SAE groups, respectively; (**h**) cell viability of the control, MXene, PSBMA, and SAE groups; (**i**,**j**) skin-electrode interfacial impedance of the SAE, ${\mathrm{Ti}}_{3}$$C_{2}T_{x}$ aerogel, and Ag/AgCl electrode before and after sweating, respectively; and (**k**) variation in skin-electrode interfacial impedance during prolonged wear. Adapted from Ref. [101].

The importance of interfacial stability becomes more pronounced during exercise, where repeated skin deformation and sweat accumulation continuously perturb electrode contact. Under these conditions, electrophysiological signal quality is influenced not only by electrode conductivity, but also by the ability of the interface to maintain low and stable contact impedance during motion. Cheng et al. identified conformal contact and signal-to-noise stability as key requirements for flexible surface-EMG electrodes [100]. Shi et al. further addressed sweat-induced interface variation using a ${\mathrm{Ti}}_{3}$$C_{2}T_{x}$/zwitterionic-polymer electrode whose adhesion and electrical coupling improved after sweat absorption [101]. Figure 6 combines adhesion measurements with impedance spectra before and after sweating and during prolonged wear, showing that sweat absorption can strengthen interfacial coupling while lowering skin-contact impedance. This result highlights a broader shift from passively resisting sweat toward designing nanomaterial/polymer interfaces that adapt to it, suggesting that impedance stability and signal fidelity under dynamic exercise conditions are more informative evaluation criteria than conductivity measured at rest.

### 3.4. Electrochemical and Self-Powered Sensing

Electrochemical wearables have evolved from controlling electrode reactions to regulating the complete pathway from biofluid sampling to molecular recognition and electrical readout. Early studies on electrically wired enzymes and impedance biosensing established the importance of electron transfer and interfacial selectivity [102,103], and these principles were subsequently translated to mechanically compliant wearable electrochemistry [104]. On-body sensing progressed from lactate and sodium monitoring [105,106] to ${\mathrm{Ca}}^{2+}$/pH, glucose, and multiparameter sensing platforms [107,108,109,110]. As device integration advanced, sampling became an integral part of transduction. Gao et al. incorporated multiplexed sweat analysis [10], Koh et al. controlled sweat capture through soft microfluidics [111], and Alizadeh et al. validated electrolyte monitoring during exertion [11]. In parallel, material chemistry and fluid regulation became increasingly coupled. Graphene-based enzyme transfer and pH sensing [112,113], quasi-two-dimensional metal oxides [114], and flexible rGO or PEDOT:PSS/porous-graphene electrodes [60,67] improved interfacial transduction, while osmotic extraction, porous interfaces, and selective nanochannels enhanced sample access and interferent control [115,116,117,118]. Electrochemical performance consequently depends on the stability of the full measurement pathway, including sample transport, molecular recognition, interfacial charge transfer, and reference conditions [119,120,121,122].

Self-powered sensing introduces an additional requirement by coupling signal acquisition with energy availability. Textile and stretchable biofuel cells harvest biochemical energy from sweat [123,124], whereas triboelectric systems convert repetitive body motion into electrical output [125]. More recent designs integrate sensing with energy storage or hybrid mechanical harvesting [126,127,128]. Although these strategies can reduce battery dependence, peak power output alone does not represent practical autonomy because signal conditioning, storage, and wireless communication impose different and time-dependent energy demands [129]. The performance of self-powered wearables is therefore better assessed through sustained energy balance over realistic duty cycles, including periods of low activity or limited sweat production, together with the stability of the sensing function itself.

The mechanisms discussed above operate over different mechanical, temporal, and biochemical ranges, and their limitations become more apparent during repeated on-body use. Hysteresis, interfacial drift, sampling variability, and power demand may dominate over nominal sensitivity when measurements leave controlled laboratory conditions. Mechanism selection should therefore be based on the deformation mode, signal timescale, body environment, and information required by the application. Across modalities, reproducibility of the relationship between stimulus and measured output remains the common requirement.

## 4. From Nanomaterial-Enabled Wearable Signals to Sports and Rehabilitation Assessment

Sports and rehabilitation provide a practical test of whether material-level sensing advantages translate into reliable measurements during real use. High surface area, percolated conductive networks, and catalytic interfaces are valuable only if body coupling, sweat sampling, and calibration remain sufficiently stable for the measured signal to track the intended biomechanical or physiological variable. The following applications are therefore considered in terms of both sensing capability and the reliability of the information obtained.

### 4.1. Joint Motion, Gait, and Biomechanical Loading

For biomechanical monitoring, the objective has progressively shifted from detecting the occurrence of movement to quantifying motion with sufficient mechanical specificity. Hwang et al. used a self-powered stretchable platform for human-activity recognition [130], whereas Geng et al. related capacitive strain to joint angle [131]. Breathable strain sensors now extend this mapping to prolonged joint and gesture monitoring [26]. At this stage, the sensitivity of a nanostructured active layer is only one part of the measurement chain: attachment geometry and strain transfer determine how faithfully joint deformation reaches that layer. The same electrical response may therefore correspond to different joint motions when sensor placement or local skin deformation changes.

**Figure 7:**
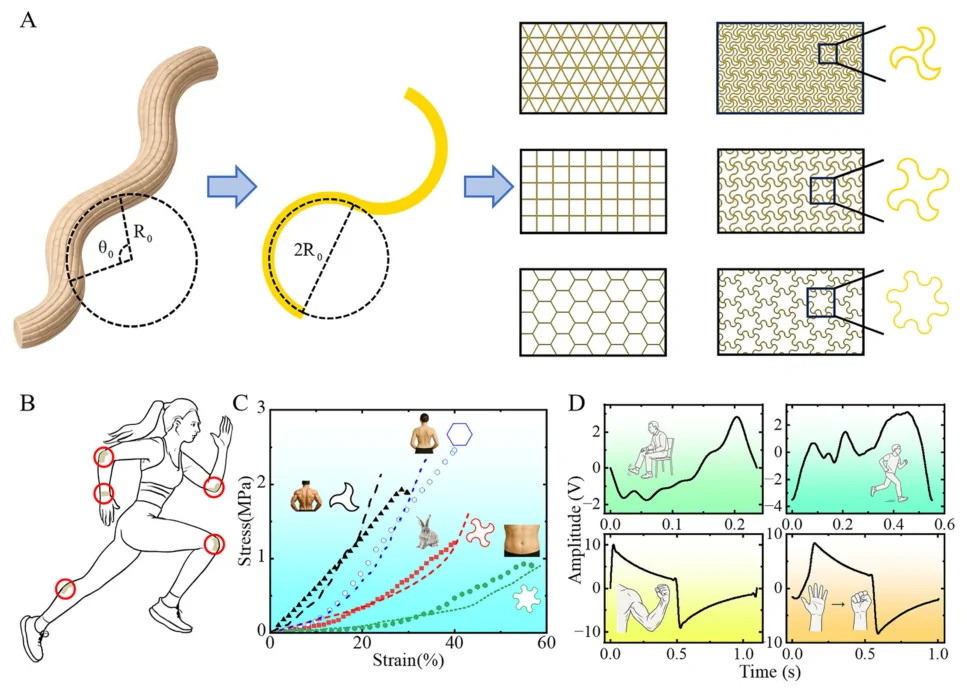
Metamaterial-inspired flexible sensor for biomechanical monitoring: (**A**) structural design of the metamaterial-inspired sensor; (**B**) representative sensor placement on the human body; (**C**) mechanical response and skin-matching characteristics under deformation; and (**D**) motion-dependent electrical responses of the sensor. Adapted from Ref. [133].

Self-powered sensing poses the same mechanical-coupling problem. Hydrogel triboelectric sensors provide compliant motion-dependent output [132], while Kong et al. used a metamaterial-inspired flexoelectric lattice to match local skin mechanics [133]. Figure 7 relates lattice geometry and body placement to strain response and electrical output, showing how structural design affects the transfer of local deformation to the active sensing element. For joint or gait assessment, repeatable coupling between body motion and sensor deformation is more informative than output amplitude alone.

### 4.2. Physiological Monitoring During Exercise

**Figure 8:**
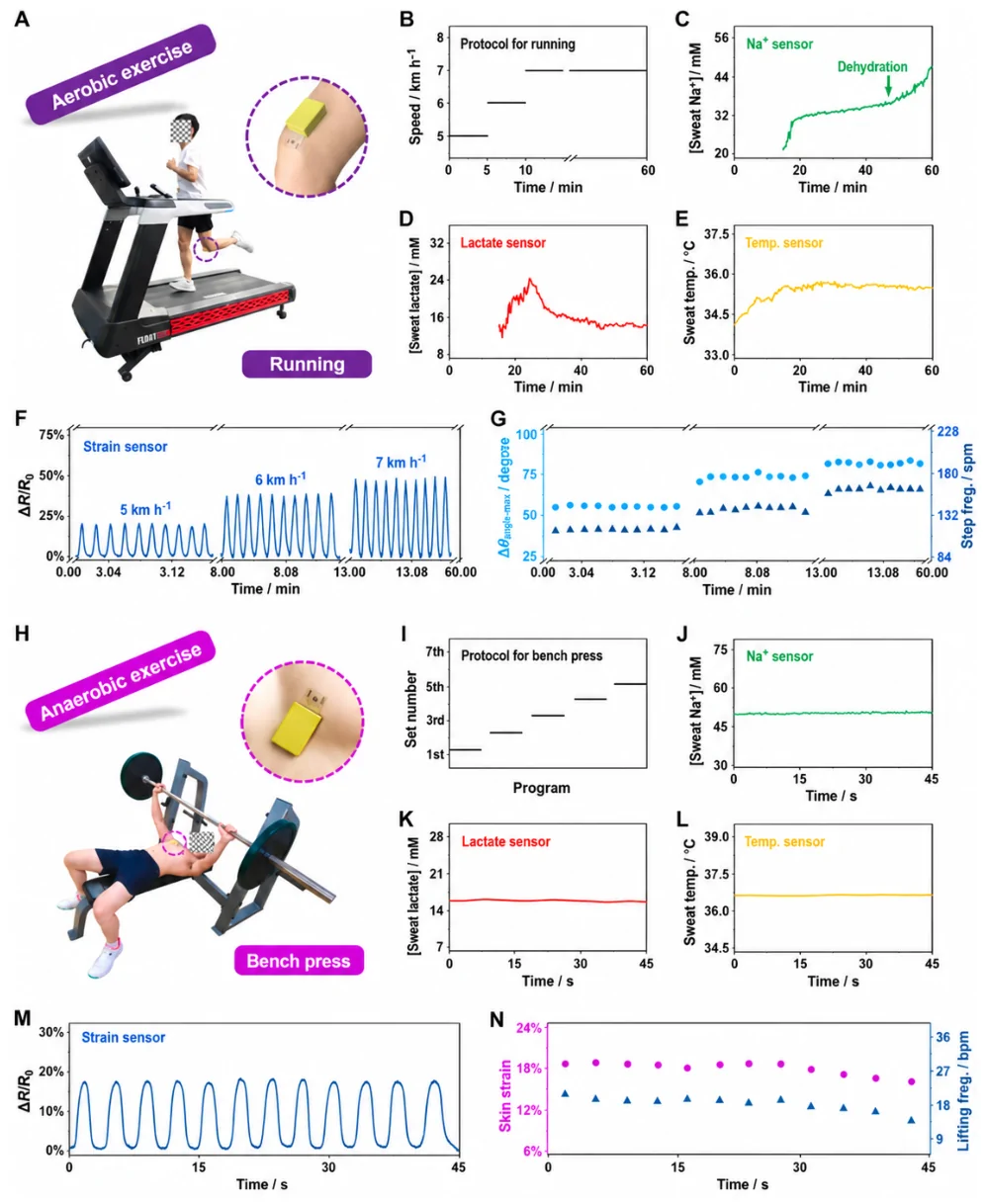
Simultaneous biomechanical and metabolic monitoring using the iTrainer platform: (**A**) wearable sensing configuration during aerobic running; (**B**) running protocol; (**C**) sweat ${\mathrm{Na}}^{+}$ response; (**D**) sweat lactate response; (**E**) sweat-temperature response; (**F**) strain-sensor response at different running speeds; (**G**) corresponding knee-angle variation and step frequency; (**H**) wearable sensing configuration during anaerobic bench press; (**I**) bench-press protocol; (**J**) sweat ${\mathrm{Na}}^{+}$ response; (**K**) sweat lactate response; (**L**) sweat-temperature response; (**M**) strain-sensor response during repeated bench press; and (**N**) corresponding skin strain and lifting frequency. Adapted from Ref. [136].

Physiological monitoring adds information that motion signals alone cannot provide. Skin-mounted transcutaneous oxygen sensors first demonstrated continuous acquisition of a physiological variable [134], and Imani et al. later integrated electrophysiology with sweat chemistry [99]. Reviews focused on athlete monitoring now position such channels within exercise-specific assessment [135]. Cai et al. further developed iTrainer for simultaneous skeletal–muscular and metabolic monitoring during aerobic and anaerobic exercise [136]. Figure 8 shows the exercise protocols together with synchronous strain, sweat ${\mathrm{Na}}^{+}$, lactate, and temperature channels, making it clear that mechanical and biochemical signals operate on different amplitudes and timescales but can provide complementary context. Multimodality is therefore useful when distinct material or interfacial sensing elements contribute nonredundant physiological information, not simply when sensor count increases.

Cross-modal interpretation remains sensitive to unequal response times, interface drift, and sampling delay. Mechanical and electrophysiological signals can change rapidly, whereas sweat biomarkers are additionally governed by secretion and transport lag. Synchronized acquisition requires shared timestamps or a common reference clock, with channel-specific sampling rates and response delays accounted for before signals are aligned. Co-located sensors may suffer from electrical crosstalk through shared traces and readout electronics, while cross-sensitivity arises when one stimulus affects more than one sensing element. Mu et al. demonstrated reduced cross-sensitivity through vertically integrated, modality-selective sensing structures [137]. Electrical isolation and independent channel testing help identify and limit these interference effects. Data fusion may combine calibrated features at the signal or decision level, while machine-learning methods can assist in multichannel pattern recognition [138]. Unstable or unsynchronized inputs can nevertheless limit the physiological meaning of the resulting model. Motion artifacts and electrical noise can generate spurious features that models may associate with particular exercises rather than the underlying movement. Baseline drift shifts the distribution of input features between training and later sessions, while inconsistent calibration across devices or users can make hardware-dependent offsets appear predictive of physiological differences. As a result, apparently strong within-session classification may not generalize to new sessions or participants. Signal-quality screening, drift-aware calibration, and evaluation across independent sessions and devices are therefore needed before model performance can be considered reliable. Reliable data fusion depends on modality-specific calibration and temporal alignment.

### 4.3. Sweat Chemistry, Hydration, and Fatigue-Related Indicators

**Figure 9:**
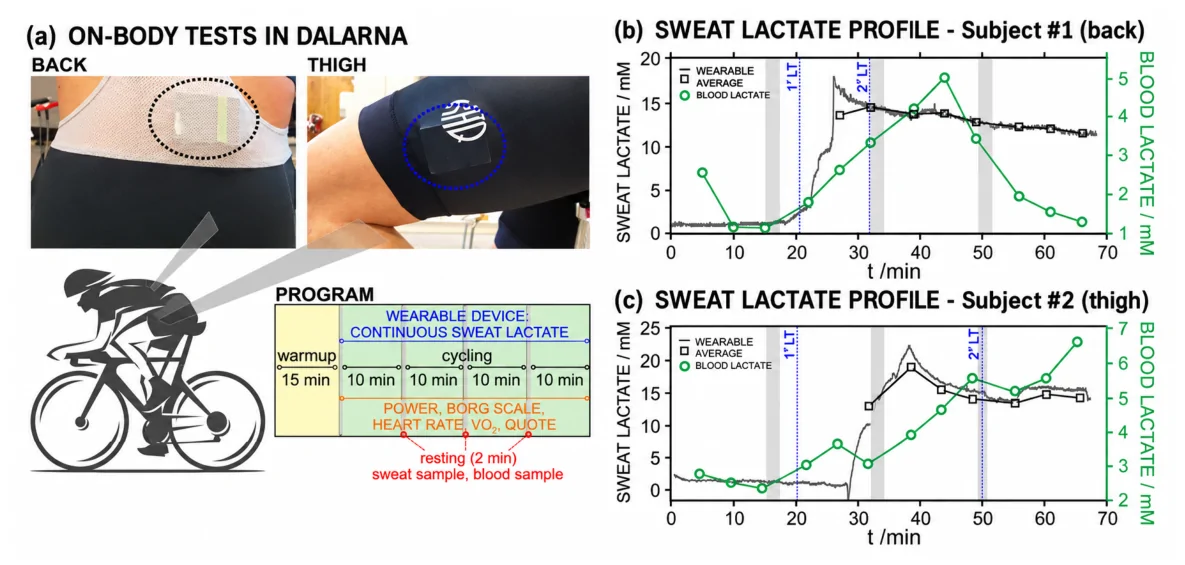
Continuous sweat-lactate monitoring during cycling: (**a**) on-body sensor placement on the back and thigh together with the cycling protocol; (**b**) sweat-lactate profile measured from the back of Subject 1 and comparison with blood lactate; and (**c**) sweat-lactate profile measured from the thigh of Subject 2 and comparison with blood lactate. Adapted from Ref. [139].

Sweat sensing illustrates the difference between analytical detection and physiological interpretation. Xuan et al. showed that lactate profiles measured at the back and thigh differed during the same cycling protocol [139]. Figure 9 compares the two on-body measurements and shows distinct temporal responses. These results indicate that local secretion, sampling location, and muscle activity can alter the measured biochemical profile even when the sensing chemistry is unchanged. Absolute concentrations must be interpreted together with the sampling site and temporal context.

Recent sweat-sensing systems incorporate sampling conditions more explicitly into analysis. Textile chloride/pH platforms provided an early route to multiparameter sensing [140], while Janus textiles added control over sweat transport and volume [141]. Zhong et al. combined phenylalanine, chloride, and sweat rate to reduce interindividual ambiguity [142], and athlete-oriented platforms have integrated electrolytes, lactate, and sweat-rate monitoring [143]. Microfluidic electrochemical/colorimetric systems further coordinate directed sweat transport with metabolite and skin-environment measurements [144], while battery-free lactate sensing and combined electrolyte-pulse monitoring extend these measurements toward fatigue and recovery assessment [145,146]. In these systems, analyte concentration is interpreted together with the conditions under which the sample is generated and collected.

For exercise applications, reproducible temporal trends are often more informative than fixed biomarker thresholds. Sweat composition varies with sampling site, secretion dynamics, fluid transport, exercise intensity, and individual baseline, so measured concentration does not necessarily reflect systemic physiological status in a unique way. Sampling location is an important consideration because regional differences in sweat gland activity and local skin conditions can affect both sweat composition and the amount of fluid available for analysis. Sweat secretion rate also influences analyte concentration and sample renewal, meaning that changes in measured concentration may result from variations in secretion or dilution rather than changes in the underlying physiological state. Furthermore, analytes must travel from the skin surface to the sensing interface through the collection pathway, where transport distance, residence time, and residual sample volume may introduce response delays or attenuate short-term concentration changes [12,13]. These effects complicate the interpretation of transient physiological responses during exercise, when sweating dynamics and metabolic demands change over time. Zhong et al. combined measurements of sweat phenylalanine, chloride, and sweat rate to investigate interindividual differences during exercise, illustrating the importance of considering secretion dynamics alongside analyte concentrations [142]. Individual baselines and differences in sweat production may also affect the comparability of measurements across users, particularly in hydration and fatigue assessment. Consequently, analytical sensitivity and the detection limit should be distinguished from physiological relevance. Although these analytical metrics characterize a sensor’s ability to detect an analyte, they do not establish whether the measured signal reliably reflects a meaningful physiological change. This requires validation against appropriate reference measurements and consideration of sampling conditions, temporal variation, and individual baselines. Parrilla et al. compared on-body sweat-ion measurements with ion chromatography and pH measurements, illustrating the importance of validating wearable sensor outputs against established analytical methods [12]. For practical applications, sweat-based sensing should therefore account for sampling location, secretion rate, and analyte transport, while emphasizing longitudinal measurements and within-subject trends. Where appropriate, complementary physiological variables should also be incorporated to support the interpretation of hydration status, fatigue, and other exercise-related responses.

### 4.4. Rehabilitation and Closed-Loop Functional Assessment

Rehabilitation requires measurements to remain comparable across repeated sessions so that changes in movement quality can be distinguished from variations in sensor performance. Textile capacitive monitoring and self-powered interaction systems have established wearable pathways for tracking therapeutic exercises [147,148]. For nanomaterial-enabled devices, stable conductive networks, durable interfaces, and repeatable strain transfer are essential, as material drift or altered contact can otherwise be misinterpreted as changes in patient performance. Feedback systems introduce an additional requirement: calibration must remain stable despite repeated attachment, deformation, and perspiration.

**Figure 10:**
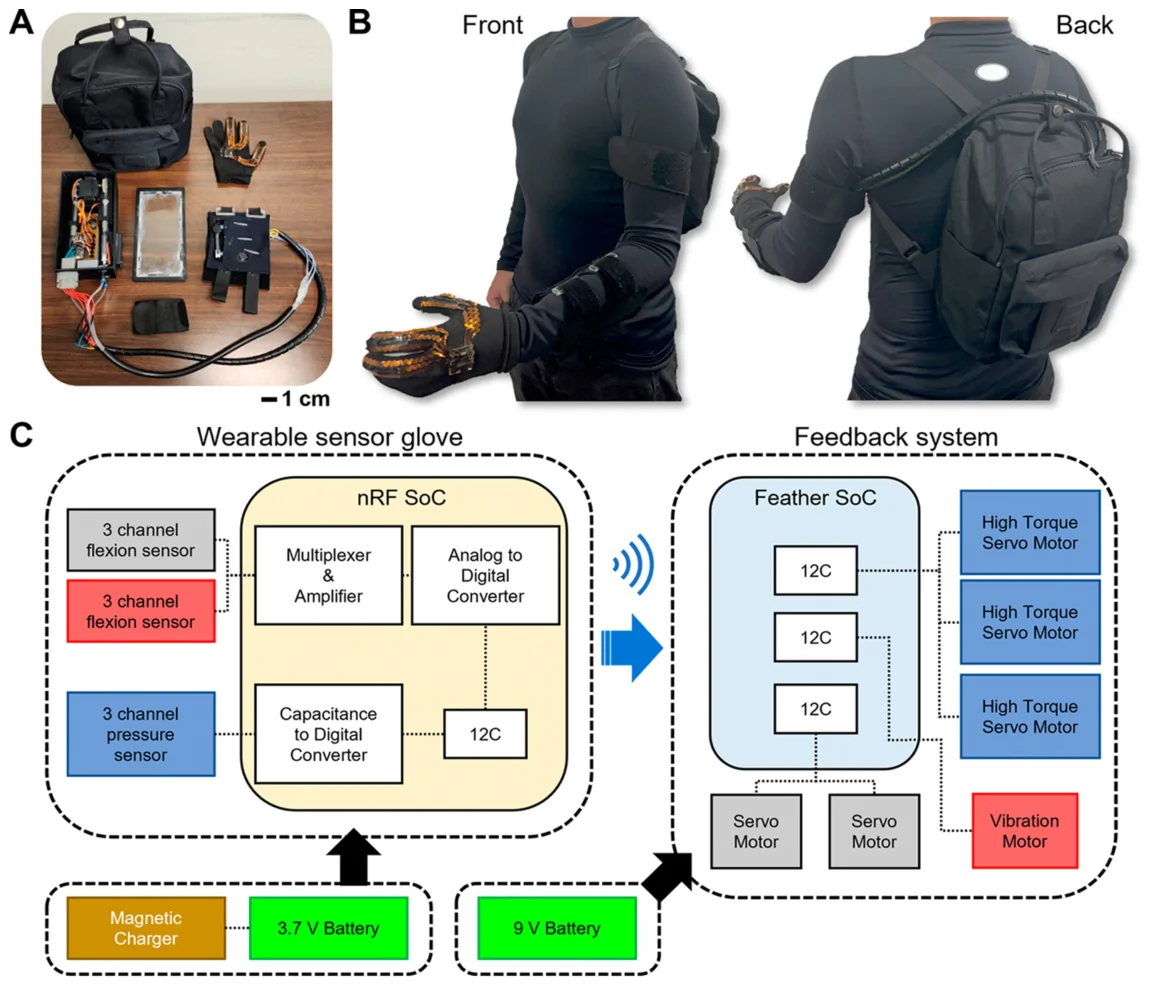
Wearable multimodal sensing and feedback system for upper-limb rehabilitation: (**A**) components of the wearable sensing and feedback platform; (**B**) front and back views of the body-worn system; and (**C**) system architecture showing multimodal sensing, signal acquisition, wireless communication, and tactile-feedback control. Adapted from Ref. [149].

Kang et al. integrated pressure, temperature, and finger-flexion sensing with wireless tactile feedback in a wearable glove [149]. Figure 10 shows the body-worn hardware alongside the sensing and feedback pathway, illustrating how measured signals are incorporated into closed-loop assistance. Robust triboelectric webbing extends this concept to embedded self-powered rehabilitation sensing [86]. The primary criterion is whether sensing and feedback remain consistently coupled across repeated therapeutic use.

Sports and rehabilitation studies increasingly seek quantitative and physiologically interpretable measurements rather than simple event detection. The examples reviewed in this section show that application performance remains closely tied to material and interface behavior. Joint-motion sensing requires repeatable transfer of deformation to the active network; multimodal monitoring depends on stable, synchronized interfaces; and sweat analysis requires controlled sampling, as well as selective electrochemical transduction. Rehabilitation adds the need for comparability across sessions. Many current demonstrations remain short-term or task-specific; therefore, calibration stability, device-to-device reproducibility, and transferability across users remain important barriers to deployment.

## 5. Reliability, Integration, and Future Perspectives

The translation of wearable sensing beyond proof-of-concept devices depends on measurement consistency across the full path from material response to physiological interpretation. Calibration drift, variable body contact, environmental exposure, and fabrication variability can all alter sensor output during repeated use. These effects are particularly important in sports and rehabilitation because meaningful comparisons across sessions or users require stable measurement conditions.

### 5.1. Material and Interface Reliability Under Dynamic Conditions

Reliability under dynamic conditions is closely related to the mechanisms used to obtain high sensing sensitivity. Near-threshold percolation, microcracks, and localized conductive contacts can amplify small mechanical inputs, but the same structural evolution may narrow the working range and increase hysteresis or baseline drift. In contrast, more redundant conductive networks generally improve mechanical robustness and recovery, although this can reduce the magnitude of the sensing response [3,49]. These trade-offs make it necessary to evaluate sensitivity together with parameters that describe measurement stability. Hysteresis can be quantified from the maximum difference between loading and unloading signals normalized to the full-scale output, while baseline drift describes the change in the unloaded signal over a specified period. Repeatability can be evaluated using the coefficient of variation under repeated identical loading conditions, and response time should be reported together with a clearly defined response criterion and loading rate. Long-term signal stability can be further characterized by changes in sensitivity and baseline after specified cycling or wear periods. Liu et al. reported a carbon nanotube-based strain sensor with 5% hysteresis, a response time of 62 ms, and repeatable performance over 10,000 cycles [150]. Yuan et al. reduced baseline drift through interfacial design in CNT-PDMS/Ag strain sensors, achieving 99.999% resistance recovery and stable operation over 5000 cycles [151]. These results illustrate the value of quantitative stability metrics, but cyclic testing under a single mechanical condition does not by itself establish reliability during practical wearable use.

The interpretation of these metrics becomes more complex when mechanical loading is coupled with environmental and interfacial changes. MXenes are susceptible to oxidation, hydrogels may undergo solvent loss, and conductive composites can gradually rearrange during repeated deformation, while excessive encapsulation may improve material protection at the expense of breathability and conformability [8]. Sweat and humidity can modify interfacial impedance, ionic transport, and conductive pathways, whereas repeated rubbing or attachment can damage conductive coatings and alter contact with the skin. These effects may change not only the baseline signal but also the apparent sensitivity and recovery behavior measured during use. Chen et al. demonstrated breathable epidermal electrodes that supported EMG recording during 5 h of sweating and 7 days of wear, highlighting the importance of moisture management for prolonged skin attachment [152]. For reusable textile sensors, Bae et al. reported only 1.40% signal variation after 1000 bending cycles, whereas the pressure response decreased by up to 38% after 10 washing cycles because of coating detachment and abrasion [153]. Luo et al. further evaluated sweat-sensing yarns under cyclic rubbing and 36 h of on-body wear, demonstrating that friction resistance and prolonged interface stability are relevant to sustained sensing performance [154]. The contrast between mechanical cycling and environmental exposure indicates that durability obtained under one loading mode cannot necessarily be extrapolated to realistic wearable conditions. Reliability assessment should therefore consider calibration retention, signal drift, and reproducibility after combined exposure to sweat, humidity, repeated deformation, washing, abrasion, and prolonged attachment.

A more useful basis for design optimization is therefore to evaluate sensing performance and stability within the operating conditions required by the intended application rather than ranking devices according to initial sensitivity alone. For mechanical sensors, gauge factor should be considered over the intended strain range together with normalized hysteresis, baseline drift, response time, and cyclic stability. A high gauge factor provides limited practical benefit if it is confined to a deformation range narrower than that encountered during joint or muscle motion, or if the associated signal exhibits substantial hysteresis after repeated loading. For biochemical sensors, calibration sensitivity and detection limit should likewise be interpreted together with sweat-dependent signal variation, sampling delay, and calibration stability [12]. An increase in analytical sensitivity does not necessarily improve physiological measurement if changes caused by secretion or sampling are comparable to the analyte variation in interest. Sensitivity retention after a defined mechanical or environmental exposure can be expressed as $100(S_{\mathrm{after}}/S_{\mathrm{before}})\%$, where *S* represents the gauge factor or calibration slope measured over the same operating range. Reporting this value together with strain amplitude, loading frequency, cycle count, exposure duration, and sweat or humidity conditions provides a quantitative basis for comparing changes in sensing performance under relevant operating conditions. Rather than assigning universal thresholds, acceptable levels of hysteresis, drift, and sensitivity loss should be determined according to the measurement accuracy and temporal resolution required for a specific application [3,155]. Material and interface design can then be evaluated according to whether improvements in sensitivity are retained without introducing unacceptable losses in working range, stability, or environmental robustness.

### 5.2. System Integration, Calibration, and Translation

The transition from single-modality sensing to integrated physiological and biomechanical monitoring increases both information content and system complexity. Mechanical, electrophysiological, thermal, and biochemical channels differ in response time, sampling frequency, calibration requirements, and susceptibility to motion or environmental interference. Hybrid systems demonstrate that multiple sensing functions can share a common soft interface [99,156], but additional channels also increase the demands on synchronization, interconnection, and cross-modal calibration. Because these modalities operate over different temporal and physical scales, their signals cannot be combined reliably without accounting for channel-specific response delays, sampling schedules, and interface conditions. Mechanical and electrophysiological signals may respond almost immediately to movement, whereas biochemical measurements are additionally influenced by secretion, sample transport, and analyte diffusion. Shared timestamps or a common reference clock can improve temporal alignment, but the intrinsic response characteristics of each sensing channel must still be considered before multimodal data are fused.

System-level performance further depends on energy availability, fabrication reproducibility, and data processing. Mechanical and biochemical energy harvesters can reduce battery dependence [124,157], although sustained operation must account for signal conditioning, storage, and wireless communication [129]. Chung et al. integrated a skin-conformal thermoelectric generator with graphene/Au/Pt-modified microneedles for continuous interstitial-fluid glucose monitoring, illustrating the potential to combine power delivery with stable body-interfaced sensing [158]. However, integration on a common substrate may also introduce electrical crosstalk, thermal interference, and incompatibilities between sensing interfaces. These effects become increasingly important as the number of sensing channels increases and may affect both individual signal quality and subsequent physiological interpretation.

Scalable printing and digital manufacturing support device fabrication, but variations in material deposition and interface formation can translate into device-to-device differences in sensitivity and calibration [159]. Such variations may arise from differences in nanomaterial dispersion, ink formulation, coating thickness, electrode geometry, curing conditions, and encapsulation. In nanomaterial-based sensors, even modest deviations in these parameters can alter conductive-network connectivity, contact resistance, strain transfer, or the effective electrochemical interface. Their influence may therefore extend beyond initial sensitivity to baseline output, hysteresis, response time, and signal drift during repeated operation. Batch-to-batch variation is particularly relevant because good repeatability within a single device does not demonstrate reproducibility across independently fabricated devices or production runs.

Fabrication reproducibility should therefore be assessed using devices prepared independently and, where possible, from multiple batches under consistent testing and calibration conditions. In addition to representative values, the distribution of sensitivity, working range, hysteresis, baseline drift, and performance retention should be reported to quantify device-to-device variation. Calibration consistency warrants separate consideration. A shared calibration curve is advantageous for practical deployment, whereas substantial differences between devices may require individual calibration and increase production and maintenance costs. Comparing shared and device-specific calibration performance can help determine whether observed variations can be compensated through calibration or instead reflect structural inconsistencies that require improved process control. Manufacturing tolerances and quality-control criteria should consequently be defined in relation to the measurement accuracy required for the intended application as fabrication moves toward larger-scale production.

Data-driven methods may assist personalization and multimodal interpretation [29], but their value remains contingent on stable interfaces and reproducible input signals. This requirement is particularly important for machine-learning-based analysis. Baseline drift, device-specific offsets, and inconsistent calibration can change the statistical distribution of input features between training and later use, potentially causing models to learn hardware- or session-specific characteristics rather than the underlying physiological response. Signal-quality screening, drift-aware calibration, and validation across independent devices, sessions, and users are therefore important for evaluating model generalizability. Multimodal data fusion and machine-learning-assisted signal processing have been demonstrated in research prototypes [138,160], whereas generalizable AI-assisted calibration and autonomous self-calibration during prolonged wear remain less mature and require further validation.

Integrated wearable systems must preserve measurement consistency despite interactions among materials, interfaces, power supply, fabrication, and signal processing. Sharing a substrate and readout electronics can simplify packaging, but it also couples previously independent sources of error. Electrical isolation, channel-specific calibration, and cross-channel validation are needed to distinguish physiological changes from correlated interference, sensor drift, or changes in interface conditions. In this context, system integration should be assessed not only by the number of sensing functions included in a platform, but also by whether those functions retain their individual measurement fidelity after integration.

Energy management introduces an additional system-level trade-off. Continuous high-rate acquisition, on-device processing, and wireless transmission increase power demand, while aggressive duty cycling can reduce temporal resolution and interrupt the acquisition of short-lived physiological events [160]. Energy harvesting can partially offset this demand, but the available power may vary with movement, environmental conditions, and operating time. Energy storage can buffer intermittent supply, although practical autonomy depends on the balance among harvested energy, conversion losses, storage capacity, and the average power consumption of the complete platform. For multimodal systems, these constraints are especially relevant because different channels may require different sampling rates while maintaining sufficient temporal correspondence for data fusion. Power performance should therefore be evaluated together with measurement continuity rather than independently of sensing and communication requirements.

Practical translation further requires coordinated consideration of manufacturing quality, calibration transfer, and application-specific validation. At production scale, batch variability should be evaluated against predefined tolerances for calibration error, device yield, and performance retention. Standardized benchmarking should include synchronization accuracy, cross-talk, baseline drift, device-to-device variability, calibration transfer, and stability under representative mechanical and environmental conditions. The required acceptance criteria will depend on the intended use of the device, since general sports monitoring, rehabilitation assessment, and clinical decision support impose different levels of measurement accuracy and risk. Validation should therefore follow applicable ISO standards and relevant regulatory requirements according to the intended application, while devices intended for clinical use additionally require appropriate clinical and longitudinal validation against reference measurements.

These considerations indicate that the translation of integrated wearable sensors cannot be judged solely by the performance of an individual material, sensing element, or prototype. Reliable operation requires consistency across fabrication, calibration, signal acquisition, energy supply, and data interpretation. Ultimately, the practical value of a wearable platform depends on whether the relationship between physiological or biomechanical changes and recorded signals remains reproducible across devices, users, sessions, and realistic operating conditions.

## 6. Conclusions

In summary, nanomaterial-enabled wearable sensors provide a practical route to achieve continuous biomechanical and physiological monitoring during sports and rehabilitation. The literature reviewed here shows that advances in nanoscale material design have improved sensitivity, compliance, and functional integration, but these gains do not by themselves ensure reliable on-body measurement. Signal quality depends on how material structure and interfacial behavior affect transduction during repeated deformation, perspiration, and prolonged contact with the body. Viewed across the full measurement chain, stable sensing requires the material response to remain reproducibly linked to the physiological or biomechanical variable of interest. Future work should therefore strengthen interface stability and device reproducibility while evaluating AI-assisted calibration, self-calibrating operation, and multimodal interpretation through standardized benchmarks and longitudinal clinical validation. Addressing these issues is necessary if nanomaterial-enabled wearable sensors are to move from short-term demonstrations toward dependable tools for quantitative sports assessment and rehabilitation monitoring.

## Figures and Tables

**Table 1:** Representative functional nanomaterials, architectures, and performance metrics for flexible wearable sensing.

Author	Functional Composition	Nanoarchitecture/Active Interface	Transduction	Representative Performance
Yamada et al. (2011) [17]	SWCNT/PDMS	Aligned SWCNT film forming strain-induced gaps, islands, and bridging bundles	Piezoresistive strain	Strain up to 280%; 14 ms response; 10,000 cycles at 150% strain
Wang et al. (2014) [15]	Graphene woven fabric/PDMS	Interconnected graphene ribbons with strain-dependent junction resistance	Piezoresistive strain	GF ≈ 35 to 106 depending on strain regime
Shi et al. (2016) [16]	Graphene/CNT hybrid	Graphene grown within CNT-network voids to reinforce junctions and load transfer	Piezoresistive strain	Stable repeated deformation up to 20% strain
Boland et al. (2016) [34]	Graphene/polysilicone	Mobile graphene nanosheets in a viscoelastic polymer matrix	Piezoresistive strain	GF > 500; dynamic response at frequencies >160 Hz
Cai et al. (2018) [43]	${\mathrm{Ti}}_{3}$$C_{2}$$T_{x}$/CNT	2D MXene nanostacks bridged by 1D CNTs across strain-induced cracks	Piezoresistive strain	GF up to 772.6; 0.1% strain detection; strain up to 130%; >5000 cycles
Guo et al. (2019) [19]	${\mathrm{Ti}}_{3}$$C_{2}$$T_{x}$/porous paper/PLA	Porous MXene-impregnated paper with pressure-dependent contact pathways	Piezoresistive pressure	Detection limit 10.2 Pa; range to 30 kPa; 11 ms response; >10,000 cycles
Li et al. (2019) [44]	${\mathrm{Ti}}_{3}$$C_{2}$$T_{x}$/cotton textile	Dip-coated MXene network supported by a wavy textile surface	Piezoresistive pressure	3.844 ${\mathrm{kPa}}^{-1}$ below 29 kPa and 12.095 ${\mathrm{kPa}}^{-1}$ at 29 to 40 kPa; 26 ms; 5600 cycles
Xu et al. (2014) [63]	Graphene/PANI/AuNP/GOx	Hybrid catalytic interface promoting direct GOx-electrode electron transfer	Electrochemical glucose	$k_{s}=4.8$$s^{-1}$; LOD 0.6 μM; 4.0 μM to 1.12 mM
Xu et al. (2017) [64]	AuNP/PANI/carbon cloth	∼20 nm AuNPs on vertical PANI arrays (∼200 nm high)	Electrochemical glucose	150 μA ${\mathrm{cm}}^{-2}$ ${\mathrm{mM}}^{-1}$; LOD 3.08 μM; 10.26 μM to 10 mM; stable response after 300 bending cycles
Zamora et al. (2018) [57]	${\mathrm{IrO}}_{x}$/stainless-steel textile	Electrodeposited iridium-oxide pH-sensitive layer on conductive mesh	Potentiometric pH	Sensitivity ≈47.54 mV ${\mathrm{pH}}^{-1}$; pH 4 to 8
Semwal and Gupta (2019) [65]	rGO/PANI-coated optical fiber	In-situ rGO/PANI nanocomposite modifies the surface-plasmon sensing interface	Optical pH	pH 2.4–11.35; maximum sensitivity 75.09 nm ${\mathrm{pH}}^{-1}$ at pH 11.35
Wang et al. (2020) [66]	Au fiber/ion-selective membrane	Stretchable gold-fiber electrode integrated with a potentiometric pH-selective interface	Potentiometric pH	pH 4–8; sensitivity 60.6 mV ${\mathrm{pH}}^{-1}$; 2.6% sensitivity variation at 30% strain
Xuan et al. (2018) [67]	Micro-patterned rGO nanocomposite	Flexible micro-patterned rGO electrode provides a high-area electrochemical glucose interface	Electrochemical glucose	Glucose range 0–2.4 mM; sensitivity 48 μA ${\mathrm{mM}}^{-1}$ ${\mathrm{cm}}^{-2}$; LOD 5 μM; 20 s response
Manjakkal et al. (2019) [68]	Graphite composite/Ag/AgCl textile electrodes	Screen-printed graphite sensing and Ag/AgCl reference electrodes on cellulose–polyester fabric	Potentiometric pH	pH 6–9; sensitivity 4 mV ${\mathrm{pH}}^{-1}$; 5 s response; stable potential of 47±2 mV over 2000 s after washing
Gao et al. (2026) [61]	PEDOT/PVA/${\mathrm{Ti}}_{3}$$C_{2}$$T_{x}$	3D porous conductive hydrogel combining PEDOT transport and MXene electrocatalysis	Electrochemical UA	0.289 μA μ$M^{-1}$ ${\mathrm{cm}}^{-2}$; LOD 0.57 μM; 1 to 400 μM linear range

**Table 2:** Representative soft matrices and body–sensor interface architectures relevant to stabilizing nanomaterial-enabled wearable sensing, with mechanical, interfacial, and environmental performance.

Author	Soft Matrix/Interface Chemistry	Dominant Interaction or Adaptation Mechanism	Mechanical/Interfacial Metrics	Environmental or Operational Stability
Sun et al. (2012) [27]	Alginate/PAAm double-network hydrogel	Ionic alginate crosslinks dissipate energy while the covalent PAAm network maintains integrity	Stretch >2000%; fracture energy ∼9000 J $m^{-2}$	∼90% water while retaining high toughness
Keplinger et al. (2013) [28]	PAAm/NaCl ionic hydrogel	Mobile ${\mathrm{Na}}^{+}$/${\mathrm{Cl}}^{-}$ ions provide conduction while the hydrogel preserves optical transparency and deformability	98.9% visible transmittance reported for a 5.48 M NaCl hydrogel	Ionic conduction retained during large deformation
Yoon et al. (2019) [69]	Bio-based supramolecular polymer/carbon thread	Reversible hydrogen bonding restores the damaged polymer/electrode pathway	Self-healing efficiency >97% after 30 s at room temperature	$K^{+}$/${\mathrm{Na}}^{+}$ sweat sensing maintained after healing
Sanchez-Botero et al. (2023) [85]	Multilayer breathable fabric capacitor	Textile layers provide distributed strain accommodation and vapor transport	Strain >90%; cyclic stability >5000 cycles	WVTR ∼50 g $h^{-1}$ $m^{-2}$; validated at 35 °C/90% RH and after washing
Huang et al. (2024) [73]	Water/ethylene-glycol organohydrogel	Binary-solvent and polar-group regulation suppresses freezing and preserves interfacial mobility	Stretchability ∼6185%; toughness 9.2 MJ $m^{-3}$; adhesion 10.2 to 27.8 kPa	Freezing tolerance to −78°C; delay-free sensing at −40°C
Zeng et al. (2024) [74]	Lignin-modified ${\mathrm{Ti}}_{3}$$C_{2}$$T_{x}$/${\mathrm{Fe}}^{3+}$ hydrogel	Lignin/MXene/${\mathrm{Fe}}^{3+}$ dual catalytic and hybrid conductive-network design	Ductility 2139%; GF up to 2.8; sensing window 0 to 947%; 200 cycles at 200% strain	Stable operation from −70 to 50 °C
Hasan et al. (2026) [87]	Guar-gum/glycerol/ ${\mathrm{Ti}}_{3}$$C_{2}$$T_{x}$/MCC hydrogel	Glycerol suppresses freezing while cellulose reinforcement and MXene maintain mechanical and conductive pathways	Stretchability 580%; tensile strength 269 kPa	Human-motion sensing maintained under subzero conditions down to −56°C; stable over 600 cycles
Wang et al. (2024) [75]	PAAm/cellulose (PAAm/Cel)	Sweat NaCl partially dissociates PAAm/cellulose H-bonds and exposes polar groups for skin adhesion	Interfacial toughness 268.3 J $m^{-2}$ on sweaty skin versus 69.2 J $m^{-2}$ on dry skin; ionic conductivity 29.8 mS ${\mathrm{cm}}^{-1}$	Stable EMG during prolonged sweating; reusable after repeated attachment
Zhou et al. (2026) [79]	Dopamine-CNT/${\mathrm{Ti}}_{3}$$C_{2}$$T_{x}$ hydrogel	Catechol-mediated interfacial coupling improves MXene dispersion and conductive-network stability	Conductivity ∼0.37 S $m^{-1}$; stretch 1400%; toughness 14.35 MJ $m^{-3}$; GF 20.15	Stable response under large repeated deformation
Wang et al. (2026) [88]	CMC-DA/PAM + borax + ChCl/glycerol	Dynamic boronate-ester network plus covalent PAM network; modular CQD and MXene/PEDOT:PSS domains	Stretch 2778.74%; toughness 9.53 MJ $m^{-3}$; strain-sensing GF 11.24	Self-healing, tissue adhesion, and anti-freezing multimodal operation
Wang et al. (2026) [86]	Hyperelastic triboelectric webbing	Embedded textured architecture and elastic yarns distribute strain while generating triboelectric output	Strength ∼54.7 MPa; strain >400%	Electrical durability >100,000 cycles
Song et al. (2026) [26]	TPU/liquid-metal porous nanofiber membrane	Selective pressing activates liquid-metal pathways while preserving the porous fibrous scaffold	Fracture elongation 200%; sensing range to 100%; 270 ms response	WVTR 2296 g $m^{-2}$ ${\mathrm{day}}^{-1}$; air permeability 120 mm $s^{-1}$; stable over 1000 cycles at 50% strain

## Data Availability

No new data were created or analyzed in this study. Data sharing is not applicable to this article.
